# Metformin Shortens Prolonged QT Interval in Diabetic Mice by Inhibiting L-Type Calcium Current: A Possible Therapeutic Approach

**DOI:** 10.3389/fphar.2020.00614

**Published:** 2020-06-11

**Authors:** Hui Wang, Cao Wang, Yuan Lu, Yan Yan, Dongjing Leng, Shanshan Tian, Dongjie Zheng, Zhiguo Wang, Yunlong Bai

**Affiliations:** ^1^Department of Pharmacology (State-Province Key Laboratories of Biomedicine–Pharmaceutics of China, Key Laboratory of Cardiovascular Research, Ministry of Education), College of Pharmacy, Harbin Medical University, Harbin, China; ^2^Translational Medicine Research and Cooperation Center of Northern China, Heilongjiang Academy of Medical Sciences, Harbin, China

**Keywords:** metformin, QT interval, diabetes, CACNA1C, L-type calcium current

## Abstract

The incidence and mortality of cardiovascular disease in diabetic patients are 2-3 times higher than those in non-diabetic patients. Abnormal function of the L-type calcium channel in myocardial tissue might result in multiple cardiac disorders such as a prolonged QT interval. Therefore, QT prolongation is an independent risk factor of cardiovascular disease in patients with diabetes mellitus. Metformin, a hypoglycemic agent, is widely known to effectively reduce the occurrence of macrovascular diseases. The aim of the present study was to evaluate the effect of metformin on prolonged QT interval and to explore potential ionic mechanisms induced by diabetes. Diabetic mouse models were established with streptozotocin and an electrocardiogram was used to monitor the QT interval after 4 weeks of metformin treatment in each group. Action potential duration (APD) and L-type calcium current (*I*_Ca-L_) were detected by patch-clamp in isolated mice ventricular cardiomyocytes and neonatal cardiomyocytes of mice. The expression levels of *CACNA1C* mRNA and Cav1.2 were measured by real-time PCR, western blot and immunofluorescence. A shortened QT interval was observed after 4 weeks of metformin treatment in diabetic mice. Patch-clamp results revealed that both APD and *I*_Ca-L_ were shortened in mouse cardiomyocytes. Furthermore, the expression levels of *CACNA1C* mRNA and Cav1.2 were decreased in the metformin group. The same results were also obtained in cultured neonatal mice cardiomyocytes. Overall, these results verify that metformin could shorten a prolonged QT interval by inhibiting the calcium current, suggesting that metformin may play a role in the electrophysiology underlying diabetic cardiopathy.

## Introduction

Diabetes is a metabolic disease characterized by hyperglycemia with serious long-term complications including cardiovascular disease, stroke, chronic kidney disease, foot ulcers, or eye damage (Scheen, 2018). Cardiovascular complications are the main cause of death in diabetic patients, and the occurrence of a prolonged QT interval is positively correlated with the risk of sudden cardiac death (Whitsel et al., 2005; Lu et al., 2013). As a fatal arrhythmia, nearly half of the patients with type 1 diabetes have a prolonged corrected QT (QTc) interval (> 440 milliseconds) (Rossing et al., 2001). In addition, studies on patients with type 1 diabetic nephropathy showed that increased QTc was a mortality predictor (Sawicki et al., 1996). In a population-based cohort study of patients with type 2 diabetes, increased QT interval dispersion (QTd) was found to be a long-term, independent risk factor for cardiovascular death compared with other cardiovascular diseases (Giunti et al., 2012). Consequently, it is necessary to control the incidence of QT prolongation in diabetic patients to prevent the worsening of cardiac disorders.

Electrocardiogram signals reflect the action potential duration (APD) which in turn reflects the electrical activity of typical cardiomyocytes (Marbán, 2002). The QT interval represents the duration of depolarization and repolarization in ventricular myocytes (Schouten et al., 1991). A prolonged QT interval in diabetes mellitus is considered to represent an ion channel disorder involving potassium and calcium channels (Yada et al., 2007). The L-type calcium channel (LTCC) encoded by the *CACNA1C* gene is a subunit of the L-type voltage-dependent calcium channel, which mediates calcium influx into the cell membrane and provides an essential Ca^2+^trigger for excitation-contraction coupling (Eden et al., 2016). A study showed that a high concentration of glucose is necessary for promoting Cav1.2 channel activity (Nystoriak et al., 2017). Dominant augmentation of the L-type calcium current (*I*_Ca-L_) in the heart results in QT prolongation as well as APD (Yada et al., 2007; Liang et al., 2019). The remodeling of LTCC in diabetic cardiomyocytes may be one of the important mechanisms contributing to the prolonged QT interval in patients with diabetes mellitus (Lu et al., 2007; Hyltén-Cavallius et al., 2017).

Metformin is one of the most common drugs used to treat type 2 diabetes without causing significant hypoglycemia (Ferrannini and DeFronzo, 2015). Furthermore, metformin has attracted substantial attention owing to its pleiotropic function (Foretz et al., 2014; Campbell et al., 2017). The United Kingdom Prospective Diabetes Study (UKPDS) indicated that metformin had cardiovascular protective effects beyond reducing blood glucose levels (Ovalle, 2009; Rojas and Gomes, 2013; Natali et al., 2019). By further detecting AMPK activation, it was found that metformin could markedly decrease transforming growth factor-β1 production by inhibiting HNF4α, and ultimately suppressed cardiac fibrosis (Chen et al., 2018). Moreover, the positive effects of metformin have been indicated in the treatment of a prolonged QT interval in a diabetic state. Animal experiments showed that different doses of metformin had different effects on the QT interval in diabetic rats (Costa et al., 2008). In a randomized controlled study involving 30 patients with diabetes, Najeed found that metformin could reduce the QTc interval (Najeed et al., 2002). These emerging discoveries emphasize the protective role of metformin against prolonged QT interval. However, the potential treatment mechanism of metformin in treating prolonged QT interval remains unclear owing to a lack of in-depth studies. Based on these previous findings, we hypothesized that metformin could attenuate a prolonged QT interval by inhibiting the L-type voltage-dependent calcium channel, which may provide a new mechanism in which metformin improves cardiac function.

## Materials and Methods

### Animals and Treatments

All animal experiments and handling were performed in accordance with the National Guidelines for Experimental Animal Welfare (the Ministry of Science and Technology, People's Republic of China, 2006). All experimental protocols were pre-approved by the Experimental Animal Ethics Committee of Harbin Medical University, China (No. HMUIRB 20180025). Kunming (KM) mice (20 – 22 g) were obtained from the Second Affiliated Hospital of Harbin Medical University (Harbin, China). Type 1 diabetes was induced by a single intraperitoneal injection of 160 mg/kg streptozotocin (STZ; Sigma, St. Louis, MO, USA) dissolved in 0.01 mol/L citric acid solution (pH= 4.3). The control mice were injected with the same amount of solvent. After 7 days, fasting blood glucose levels were measured through tail veins using an Accu-Chek glucometer (Roche, Germany). Mice with fasting blood glucose levels over 11.1 mmol/L were selected as diabetic mice for further analysis (Zhang et al., 2018). According to the blood glucose level, the mice were divided into four groups: the control group, diabetes model group (DM group), and the diabetic with 50 mg/kg and 250 mg/kg metformin groups and began receiving the treatment for 4 weeks. Mice in the metformin groups were intragastrically administered with 50mg/kg or 250mg/kg metformin; the control and DM group mice received equivalent volumes of sterilized distilled water (Oliveira et al., 2016).

### Neonatal Mice Cardiomyocytes Culture

One to 3 day-old-neonatal KM mice were used for *in vitro* experiments. The neonatal mice were disinfected with 75% alcohol. Their hearts were harvested and placed in a dish containing Dulbecco's modified Eagle medium (DMEM; HyClone Laboratories, UT, USA). Each heart was sliced into a few pieces and added to trypsin- ethylenediaminetetraacetic acid (EDTA) solution (Beyotime Institute of Biotechnology, Jiangsu, China) for digestion. The digestive function of trypsin-EDTA was neutralized by DMEM containing 10% fetal bovine serum (Biological Industries, Kibbutz Beit Haemek, Israel) and the digestion steps were repeated until the tissue was completely digested. The cell suspension was centrifuged for 3 min to obtain granular cells, which were then resuspended in culture medium and incubated for 1.5 h under a humidified atmosphere of 5% CO_2_ at 37°C to allow for the attachment of fibroblasts. The suspended cells were then collected and cultured under the above conditions.

### Electrocardiograms

Mice were anesthetized with Avertin (Sigma-Aldrich, St. Louis, MO, USA) and fixed in the supine position. The Lead II surface electrocardiogram (ECG) was taken *via* a pair of electrodes that were connected to a BL420s multichannel recorder (TME Technology, Chengdu, China) for a continuous period of 10 min and QTc was calculated as described previously (Wang et al., 2012). In detail, the electrode needle was inserted subcutaneously into the limbs. The positive electrode was connected to the left lower limb, and the negative electrode was connected to the right upper limb. QT represents the interval from the beginning of the Q wave to the end of the T wave, while QTc was calculated with the formula, *QTc = QT/(RR/100)^1/2^* (Mitchell et al., 1998).

### *In Vitro* Experimental Designs

Metformin (Sigma, UK) was dissolved in Tyrode buffer to obtain a final concentration of 10μM and 30μM for cell treatment. All other reagents were of standard analytical grade. The cultured neonatal mice cardiomyocytes were divided into the following four groups with the following treatments described in detail previously:

Group 1: normal glucose concentration(NC), in which the cells were treated with 5 mmol/L glucose; Group 2: High glucose concentration(HG), in which the cells were treated with 33 mM D-glucose; Group 3: HG + Met (10 μM), in which the cells were treated with 33 mM D-glucose and 10 μM metformin;

Group 4: HG + Met (30 μM), in which the cells were treated with 33 mM D-glucose and 30 μM metformin (Zhang et al., 2014).

### Real-Time Polymerase Chain Reaction

Trizol Reagent (Invitrogen, CA, USA) was used to extract total RNA from the myocardial tissue and neonatal mice cardiomyocytes. The first strand of cDNA was synthesized using a ReverTra Ace^®^ qPCR RT Kit (Toyobo, Osaka, Japan) according to the manufacturer's instructions. Real-time PCR (RT-PCR) analysis was performed on an ABI 7500 Fast RT-PCR system (Applied Biosystems, Foster City, CA, USA), with *GAPDH* as the internal control. The thermal cycling conditions consisted of one cycle of 60s at 95°C and 40 cycles of 15 s at 95°C, 15 s at 60°C, and 45 s at 72°C. The 2^-ΔΔCt^ method was used to analyze the relative mRNA expression data normalized to the *Gapdh* level.

### Western Blot Analysis

Total protein was extracted from the myocardial tissue after animal sacrifice and culturing of neonatal mice cardiomyocytes. The protein samples were separated by electrophoresis on 6% sodium dodecyl sulfate (SDS) polyacrylamide gels and the proteins were transferred to polyvinylidene difluoride membranes (Millipore, Bedford, MA, USA). The samples were then incubated at 4°C overnight with primary antibodies for Cav1.2 (Alomone Labs, Jerusalem, Israel; 1:500) and GAPDH (ZSGB, Beijing, China, 1:1000). The membranes were washed three times for 10 min each time, with phosphate-buffered saline containing 0.5% Tween 20 (PBS-T) and then incubated with secondary antibodies (ZSGB, Beijing, China; 1:10000) at room temperature for 1 h. The images of the blots were captured with Odyssey^®^ Imaging System (LI-COR Biosciences, Valencia, CA). Quantity One software was used to quantify the western blot bands with GAPDH as a reference protein.

### Acute Isolation of Mouse Cardiac Myocytes

The mice were administered with 2% tribromoethanol (0.01mL/g) by intraperitoneal injection. The heart was removed after anesthesia and was immediately perfused through the aorta by a Langendorff device. The first step was to pump the heart blood with Ca^2+^-containing Tyrode's solution (in mM: NaCl 136, KCl 5.4, MgCl_2_ 1, CaCl_2_ 1.8, Hepes 10, and Glucose10, pH 7.4 adjusted NaOH). The flowing liquid was then converted to Ca^2+^-free Tyrode's solution at a constant rate until the heart was no longer pumping. The heart tissue was digested by enzyme solutions that contained collagenase (type II, 6.5 mg/25 mL) and bovine serum albumin (6.5 mg/25 mL). Finally, the ventricular tissue was removed when it was softened and was preserved in KB solution (in mM: Glutamic acid 70, Taurine 15, MgCl_2_ 0.5, KCl 30, KH_2_PO_4_ 10, Hepes 10, EGTA 0.5, and Glucose 10, pH 7.4 adjusted with KOH). The isolated cardiac myocytes were used for whole-cell patch-clamp recording or immunofluorescence analysis.

### Immunofluorescence

Ventricular myocytes acutely isolated from KM mice with Langendorff device are stored in KB solution. Cells were fixed with paraformaldehyde for 30 min and then washed with PBS. Subsequently, the cells were treated with a penetrant (0.6% TritonX-100 and bovine serum albumin) for 1 hour, covered with blocking medium (Goat serum, Boster) for 2 hours, and incubated with the following primary antibodies overnight at 4°C: rabbit anti-Cav1.2 antibody (1:200; Cat. No. ACC-003; Alomone Labs, Jerusalem, Israel) and GAPDH antibody (1:1000; Cat. No. Ab181602; Abcam). After washing steps (3×10 min in PBS), the cells were incubated with a secondary antibody (Alexa Fluor 488 Anti-Rabbit IgG, 1:500, A11034; Alexa Fluor 594 Anti-Mouse IgG,1:500, A11032) for 1 hour at 37°C. Finally, DAPI staining was performed for 8 min at room temperature. The cells were observed under an epifluorescence microscope (Zeiss, Jena, Germany) equipped with appropriate filter combinations.

### Whole-Cell Patch-Clamp Recording

The whole-cell patch-clamp recording was conducted in a mode used at room temperature. Currents were recorded in the whole-cell voltage-clamp mode, and the APD was recorded in the current-clamp mode with a MultiClamp 700B amplifier (Axon Instruments, CA, USA). Command pulses were produced using the Digital-Analog Converter (DigiData1440A, Molecular Devices, America) and pCLAMP10.4 software (Axon Instruments, Foster City, CA, USA) was used to capture the data. Patch-clamp techniques have previously been described in detail (Chen et al., 2010; Jiang et al., 2018). When the borosilicate glass electrode enters into the extracellular solution, the tip resistance value (2 - 4 mΩ) is displayed. The selected cells had a uniform cell size. The junction potentials are offset, and the position of the electrode is adjusted to form a giga-seal. By switching to the patch mode, a negative pressure suction is maintained until the resistance value is greater than GΩ. The membrane capacitance of each cell was recorded and the whole-cell capacitance and series resistance were compensated (usually 75% ~ 85%). *I*_Ca-L_ parameters were recorded at the following: -40mV for holding potential; the experimental voltage is increased from -40 mV to +50 mV at an interval of 10mV with a stimulation time of 300 ms. The APD parameters were recorded under the following condition: the signal was inputted through a 2-kHz filter (Zobel et al., 2002) and stimulated to a current of 3nA with a stimulation time of 2 ms (Schmidt et al., 2017).The current value was standardized through the whole-cell capacitance and was shown using pA/pF.

### Statistical Analysis

Results are presented as mean ± SEM and were analyzed with SPSS 13.0 software. Statistical comparisons among multiple groups were examined using analysis of variance (ANOVA) with *post hoc* contrasts by Tukey test. Graphs were generated using GraphPad Prism 8.0 software and a two-tailed *P < 0.05* was considered statistically significant.

## Results

### Metformin Provides Protection Against a Prolonged QT Interval in Diabetic Mice

**Figure 1:**
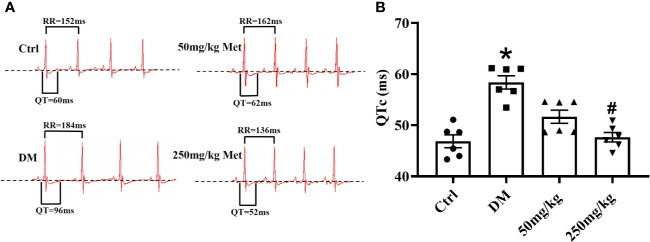
The effect of metformin on QTc in diabetic mice after 4 weeks of treatment. **(A)** Representative electrocardiogram traces recorded from surface ECG in the Ctrl group, DM group, and the metformin-treatment group for 4 weeks. **(B)** The relative level of QTc interval of the Ctrl group, DM group, and the metformin-treatment group. Values represent mean ± SEM, n_Ctrl_ = n_DM_ = n_Metformin_ = 6. *P < 0.05 *versus* Ctrl, *^#^P* < 0.05 *versus* DM.

To determine the effect of metformin against the prolonged QT interval in diabetic mice, different doses of metformin (50 mg/kg or 250mg/kg per day) or normal saline were administered to mice for 4 weeks. We first detected the effect of metformin on fasting blood glucose in diabetic mice after the 4-week treatment. As shown in **Figure S1**, there was no difference between the metformin group and DM group, which was consistent with a previous report (Abdelsamia et al., 2019). Given the evidence suggesting that HbA1c had little relevance with QTd (Syed et al.), the protective effect of metformin on LQT would not likely be dependent on a hypoglycemic effect. The electrocardiogram was monitored during the experiment. Not surprisingly, QTc was significantly prolonged in the DM group compared with that of the Ctrl group over the 4 weeks in diabetic state (**Figure 1**, **Table S1**). The lower dose of metformin treatment had no significant difference between the mice in 50 mg/kg group and the DM group. However, after intragastrical administration with 250 mg/kg metformin, the QT interval showed a progressive decline. Overall, these results indicated that 250 mg/kg metformin could alleviate the occurrence of a prolonged QT interval induced by diabetes.

### Metformin Changes the APD and L-Type Calcium Current (*I*_Ca-L_) in Cardiomyocytes Isolated From Diabetic Mice Heart

**Figure 2:**
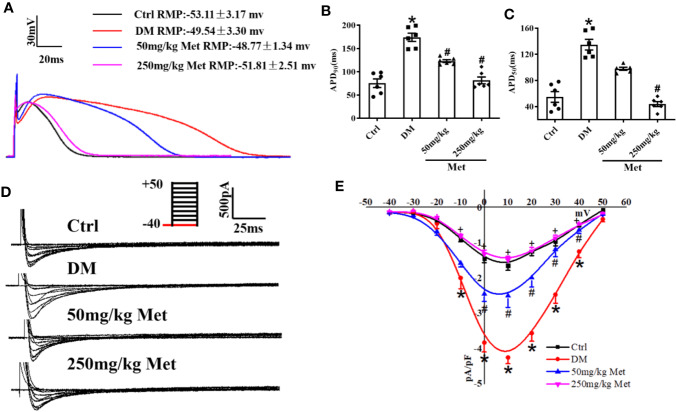
The effect of metformin on APD and *I*_Ca-L_ in cardiomyocytes isolated from diabetic mice. **(A)** Representative action potential traces in the Ctrl group, DM group, and the metformin-treatment group. **(B)** Relative levels of APD_90_ in the Ctrl group, DM group, and the metformin-treatment group. **(C)** Relative levels of APD_50_ with metformin in the Ctrl group, DM group, and the metformin-treatment group. **(D)** Representative *I*_Ca-L_ traces in the Ctrl group, DM group, and the metformin-treatment group. The holding potential was -40mV. **(E)** I-V curves of *I*_Ca-L_ in the Ctrl group, DM group, and the metformin-treatment group. Values represent mean ± SEM, n_Ctrl_ = n_DM_ = n_Metformin_ = 6. *P < 0.05 *versus* Ctrl, *^#^P* < 0.05 *versus* DM.

APD is the main factor determining the length of the QT interval. To evaluate the effect of metformin on the APD, hearts of diabetic mice were isolated and a whole-cell patch-clamp was performed to detect the APD. As expected, a significantly increased APD_90_ and APD_50_ were observed in cardiomyocytes from diabetic mice compared to the control mice (**Figures 2A–C**). Nevertheless, this effect of QT prolongation was reversed in diabetic mice treated with a high dose of metformin (250 mg/kg). *I*_Ca-L_ is one of the main inward currents in the plateau phase of the APD and plays a crucial role in managing the intracellular Ca^2+^. Metformin treatment caused a reduction in the APD_50_, indicating a reduction in *I*_Ca-L_. In adult cardiomyocytes, compared with the Ctrl group, the current density of *I*_Ca-L_ increased from - 1.64 ± 0.14 to - 4.24 ± 0.17 in the DM group, and decreased to - 2.50 ± 0.33 and - 1.49 ± 0.11 after administration of low and high dose metformin, respectively. Indeed, an increased current density of *I*_Ca-L_ was observed in the DM group, which was greatly inhibited by treatment with 250 mg/kg metformin (**Figures 2D, E**). To examine the acute effects of metformin on *I*_Ca-L_, we also detected *I*_Ca-L_ in acutely isolated cardiomyocytes of diabetic mice and then added the metformin (10μM and 30μM) into the same cells. Consistently, the results showed that the increased *I*_Ca-L_ current was greatly inhibited after 30μM metformin treatment (**Figures S2A, B**).

### Metformin Downregulates CACNA1C and Cav1.2 Expression *In Vivo*

**Figure 3:**
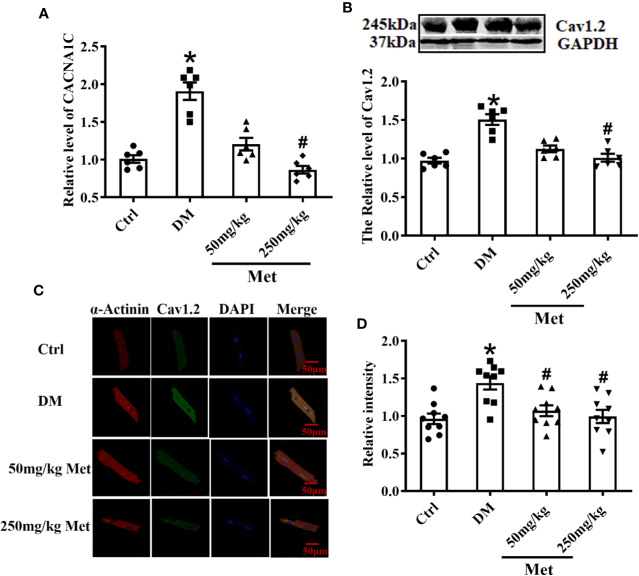
Treatment effects of metformin on the mRNA and protein levels of Cav1.2 in diabetic mice. **(A)** Relative levels of *CACNA1C* with metformin in diabetic mice detected by real-time PCR, n=6. **(B)** Relative levels of Cav1.2 in the Ctrl group, DM group, and the metformin-treatment group detected by western blot, n=6. **(C)** Representative confocal images of α-Actinin (left) or CaV1.2 (middle) in the Ctrl group, DM group, and the metformin-treatment group. **(D)** Relative levels of Cav1.2 in the Ctrl group, DM group, and the metformin-treatment group by immunofluorescences, n=9. Values represent mean ± SEM, *P < 0.05 versus Ctrl, *^#^P* < 0.05 versus DM.

As shown in **Figure 3A**, the real-time PCR results showed that metformin inhibited the expression of *CACNA1C*. In addition, the western blot demonstrated that the Cav1.2 protein level was upregulated in the DM group and downregulated in the 250 mg/kg metformin group (**Figure 3B**). Moreover, acute isolation of mouse cardiomyocytes was used to detect the expression of Cav1.2 level by immunofluorescence. It was found that the fluorescence intensity of Cav1.2 was reduced after metformin treatment (**Figures 3C, D**).

### Metformin changes the APD and L-Type Calcium Current (*I*_Ca-L_) in High Glucose-Cultured Neonatal Mice Cardiomyocytes

**Figure 4:**
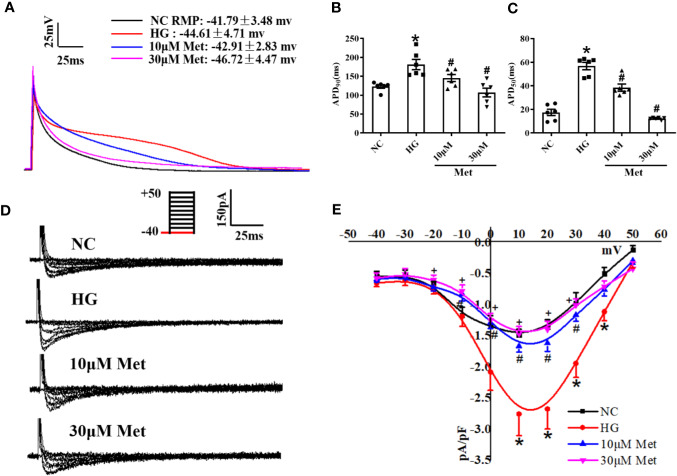
The effect of metformin on APD and *I*_Ca-L_ in high glucose cultured primary neonatal mice cardiomyocytes. **(A)** Representative action potential traces of primary cardiomyocytes in NC group, HG group and metformin-treatment group. **(B)** Relative levels of APD_90_ of primary cardiomyocytes in NC group, HG group and metformin-treatment group. **(C)** Relative levels of APD_50_ of primary cardiomyocytes in NC group, HG group and metformin-treatment group. **(D)** Representative *I*_Ca-L_ traces of primary cardiomyocytes in NC group, HG group and metformin-treatment group. The holding potential was -40mV. **(E)** I-V curves of *I*_Ca-L_, of primary cardiomyocytes in NC group, HG group and metformin-treatment group. Values represent mean ± SEM, n_NC_ = n_HG_ = n_Metformin_ = 6. *P < 0.05 *versus* NC, *^#^P* < 0.05 *versus* HG.

We further verified the results at the cellular level. Neonatal mice cardiomyocytes were treated with a high or normal concentration of glucose to imitate the diabetic condition. The cardiomyocytes were incubated for 24 h with different doses of metformin according to their groups. The single cells were digested and then the APD and *I*_Ca-L_ were detected by patch-clamp. Similar to the results for the adult mouse hearts, APD_90_ and APD_50_ were prolonged in the DM group compared with the Ctrl group and shortened after metformin treatment (**Figures 4A–C**). The *I*_Ca-L_ current density also had the same trend as the *in vivo* results. The *I*_Ca-L_ current was decreased to a further extent in high glucose-cultured neonatal mice cardiomyocytes treated with metformin. We found that the *I*_Ca-L_ current was more obviously decreased in high glucose-cultured neonatal mice cardiomyocytes with metformin. The current density of *I*_Ca-L_ increased from -1.49 ± 0.08 to -2.76 ± 0.35 after high glucose treatment and decreased to -1.66 ± 0.10 and -1.46 ± 0.12 with metformin treatment for 24 h in the low and high dose groups, respectively (**Figures 4D, E**).

### Metformin Downregulates *CACNA1C* and Cav1.2 Expression *In Vitro*

**Figure 5:**
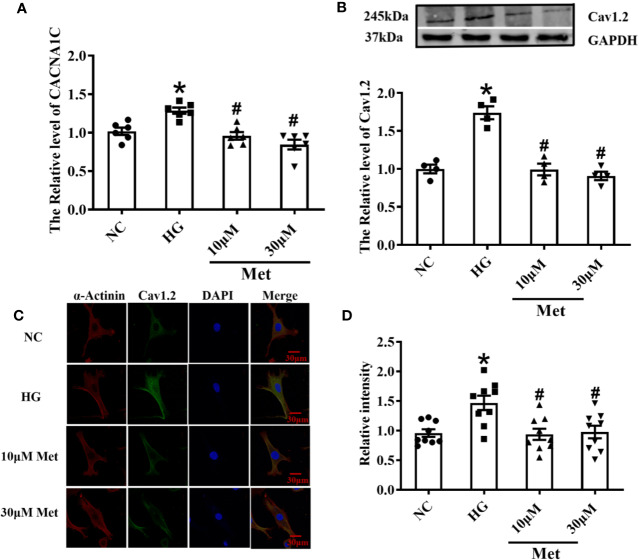
Treatment effects of metformin on the mRNA and protein levels of Cav1.2 in primary cultured neonatal mice cardiomyocytes. **(A)** Relative levels of *CACNA1C* with metformin of primary cardiomyocytes in NC group, HG group and metformin-treatment group detected by real-time PCR, n=6. **(B)** Relative levels of Cav1.2 of primary cardiomyocytes in NC group, HG group and metformin-treatment group detected by western blot, n=6. **(C)** Representative confocal images of α-Actinin (left) or CaV1.2 (middle) of primary cardiomyocytes in NC group, HG group and metformin-treatment group. **(D)** Relative levels of Cav1.2 of primary cardiomyocytes in NC group, HG group and metformin-treatment group detected by immunofluorescences, n=9. Values represent mean ± SEM, *P < 0.05 *versus* NC, *^#^P* < 0.05 *versus* HG.

Real-time PCR results showed that the increase in the *CACNA1C* mRNA level was inhibited after metformin treatment in neonatal mouse cardiomyocytes (**Figure 5A**). Furthermore, the western blot and immunofluorescence results demonstrated that the Cav1.2 protein level was upregulated in the DM group and downregulated in the metformin group (**Figures 5B–D**).

## Discussion

Recent evidence points to greater morbidity due to prolonged QT interval in diabetic patients contributing to cardiovascular death (Rossing et al., 2001). A clinical study showed that 25.6% of patients with type 1 diabetes had a QTc > 0.44 s (Sivieri et al., 1993). In the present study, we confirmed a prolonged QT interval in diabetic mice (**Figure 1**). A prolonged QT interval is highly likely to produce malignant ventricular arrhythmias, leading to a group of syndromes characterized by ventricular arrhythmia, syncope and sudden death (Wilde et al., 2016). Thus, resolving the prolonged QT interval in diabetic patients is an important goal in managing this population and preventing complications.

Metformin is an effective and safe oral hypoglycemic agent, which has been commonly used in clinical settings for more than 50 years. With an alteration in treatment patterns, a progressive protective effect of metformin on the cardiovascular system occurs *via* energy metabolism, mitochondrial energy metabolism or other pathways were increased (Griffin et al., 2017; Sun and Yang, 2017). To investigate the protective effects of metformin on the prolonged QT interval, low and high doses of metformin were administered to diabetic mice *via* oral gavage. When treated with a large dose of metformin, the QT interval was shortened in diabetic mice. The QT interval reflects the depolarization and repolarization duration of ventricular myocytes. The pathogenesis of a prolonged QT interval is believed to be congenital, or acquired by metabolic abnormalities, side effects of drugs, or an electrophysiological disorder (Moss et al., 2007; Costa et al., 2008; Arking et al., 2014). A prolonged QT interval is considered to reflect the extended APD in the ventricular myocardium (Korantzopoulos and Siogas, 2004). In the present study, we used whole-cell patch-clamp to evaluate the effect of metformin on APD. The APD_90_ and APD_50_ of mouse cardiomyocytes in the metformin-treated group were shortened compared with those of the control group. However, a lower dose of metformin reduced APD but did not influence the prolonged QTc. The membrane potential has regional variations and the QT interval depends on the longest APD in the ventricles (Wu et al., 2005). This phenomenon suggests that a lower dose metformin may not provide a sufficient intervention effect to effectively shorten the QT interval.

A prolonged action potential or QT interval is caused by a dysfunction of ion channels (including sodium, potassium, and calcium channels) or a membrane exchanger of cardiomyocytes (Matsa et al., 2014). The calcium current is the main inward current of the plateau phase and is also the main determinant of the repolarization rate of the cell membrane. Ca^2+^ is associated with various abnormalities in the ventricular myocardium, including ventricular arrhythmias and systolic dysfunction (Wagner et al., 2015). After acute isolation of the ventricular myocytes of diabetic mice, the *I*_Ca-L_ was recorded by patch-clamp, and type 1 diabetic myocytes clearly showed an increased *I*_Ca-L_ density. This finding is coincident with previous studies (Wu et al., 2020). However, other studies showed a reduced effect on the L-type calcium current in type 2 diabetic atrial myocytes or STZ-induced diabetic ventricular myocytes during 6–10 weeks (Bracken et al., 2006; Pan et al., 2015). The different influence of diabetes on *I*_Ca-L_ density is likely related to the duration and state of the disease. It may be presumed that with a longer course of diabetes, there is greater inhibition to the L-type calcium current. In the present study, *I*_Ca-L_ was distinctly suppressed in the metformin group compared to the DM group, and similar results were found in primary cultured neonatal mice cardiomyocytes. By contrast, several studies have found no effect of metformin on *I*_Ca-L_ in smooth muscle cells (Nakamura et al., 2009). This suggests a potential tissue or organ-specific effect of metformin owing to inherent features of the drug-related to its selectivity and pharmacokinetics. The discordant effect of metformin on the L-type calcium current may not only differ in terms of the different diseases but also with respect to diverse tissues and cells. It is also important however to consider the pharmacological activity of metformin on *I*_Ca-L_ in the myocardial tissue for functional selectivity in drug discovery. The present results indicated that *I*_Ca-L_ was suppressed by metformin in diabetic ventricular myocytes. Medical interventions may modify the expression and function of the L-type calcium channel and loss of Cav1.2 function may result in long QT syndrome in diabetic individuals. The molecular mechanism underlying the reduced expression of Cav1.2 with metformin treatment may involve the restoration of the physicochemical properties of the protein. However, further studies are needed to elucidate the mechanism by which metformin influences the L-type calcium channel.

In our study, there was a slight difference in the AP waveform between the mouse left ventricle cardiomyocytes and neonatal mouse cardiomyocytes; however, the *I*_Ca-L_ was similar. The AP waves were recorded from the isolated mouse left ventricle cardiomyocytes which is consistent with the present research. The neonatal mouse cardiomyocytes also showed the typical waveform (Zobel et al., 2002). The plateau phase of AP depends on the inward *I*_Ca-L_ and outward *I*_k_. Owing to the different properties of ion current in various regions of the heart, it formed the characteristic AP waveform, which may explain this difference.

**Figure 6:**
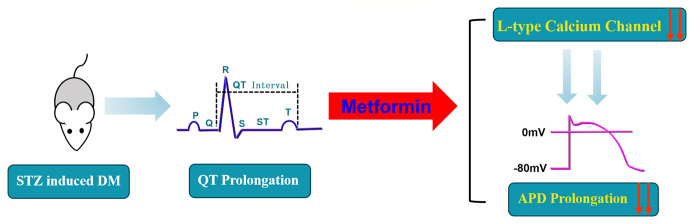
A schematic diagram of the mechanistic explanation for the effect of metformin on QT prolongation in diabetic mice.

Conclusively, our study supports the theory that a prolonged QT interval induced by diabetes could be treated by metformin through by inhibiting Cav1.2 (**Figure 6**). We discovered that metformin could affect the L-type calcium channel, which provides a novel perspective and a promising therapeutic approach for diabetes-induced prolonged QT interval.

## Data Availability Statement

The datasets analyzed in this article are publicly available. The data was uploaded as **Supplementary Material**. If need more further datasets, requests should be directed to YB, email: baiyunlong@ems.hrbmu.edu.cn.

## Ethics Statement

The animal study was reviewed and approved by Experimental Animal Ethics Committee of Harbin Medical University.

## Author Contributions

HW and YL conceptualized and designed the experiments in this study. CW performed the research and analyzed the data. YL, YY, and DL performed experimental procedures related to the study. ST, ZD and ZW proofread this article and discussed articles relating to this research. YB and ZW provided financial support. All authors contributed to the generation of experimental data for this study.

## Funding

This project was supported by the National Natural Science Foundation of China. (Grant No. 81673426 and Grant No. 81202522) and Natural Science Foundation of Heilongjiang Province (Grant No. LH2019H003).

## Conflict of Interest

The authors declare that the research was conducted in the absence of any commercial or financial relationships that could be construed as a potential conflict of interest.
