# Supplementary material for: Metformin Shortens Prolonged QT Interval in Diabetic Mice by Inhibiting L-Type Calcium Current: A Possible Therapeutic Approach
Source: Front Pharmacol. 2020 Jun 11;11:614. doi: 10.3389/fphar.2020.00614 (PMC7300225; doi:10.3389/fphar.2020.00614)

Supplementary Material

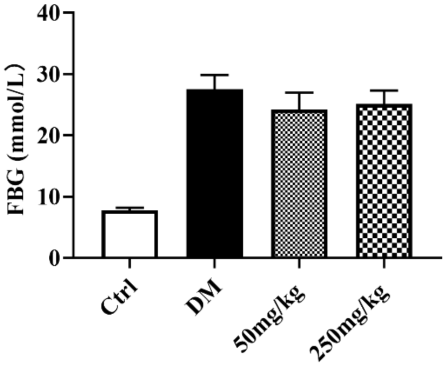

Fig.S1. The effect of FMT on hyperglycemia in diabetic mice after 4 weeks treatment. $n_{Ctrl} = n_{DM} = n_{Metformin} = 6$.

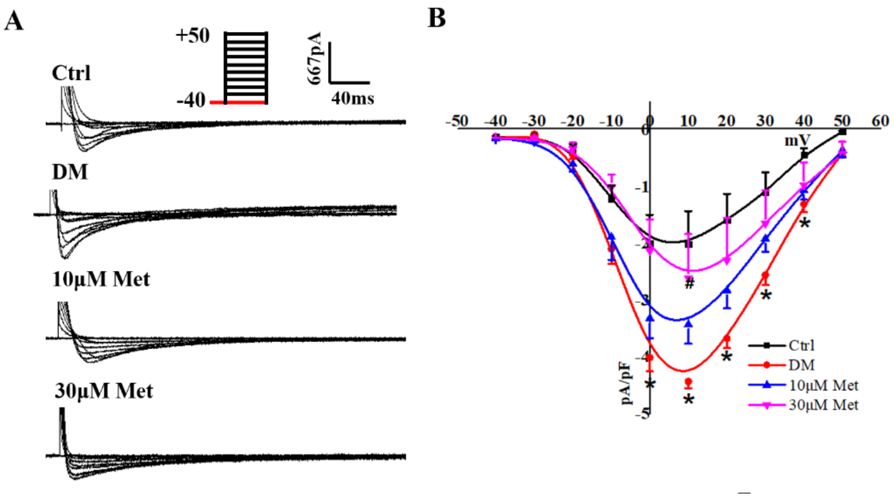

Fig.S2. The effect of FMT on ICa-L in acutely isolated cardiomyocytes of diabetic mice. (A) Representative $I_{Ca\text{-}L}$ traces in the Ctrl group, DM group, and the metformin-treatment group. The holding potential was -40mV. (E) I-V curves of $I_{Ca\text{-}L}$ in the Ctrl group, DM group, and the metformin-treatment group. Values represent mean ± SEM, $n_{Ctrl} = n_{DM} = n_{Metformin} = 6$. **P*<0.05 *versus* Ctrl, # *P*<0.05 *versus* DM.

Table S1 **The RR interval**

Group	RR interval (ms)
Ctrl	142.38±21.24
DM	156±28.59
50mg/kg Met	142.23±21.32
250mg/kg Met	141.06±24.49

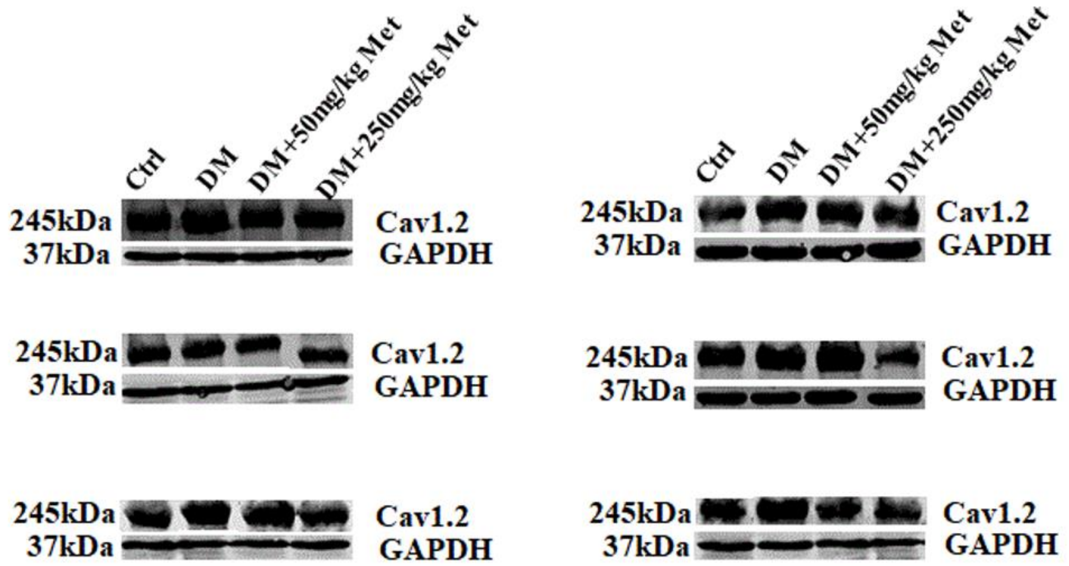

Supplement: Supplementary file 1 [file DataSheet_1.pdf]
